# AQP3 Influences Unexplained Recurrent Abortion by Regulating Trophoblast Cell Migration and Invasion via the METTL14/IGF2BP1/AQP3/PI3K/AKT Pathway

**DOI:** 10.1111/jcmm.70325

**Published:** 2025-01-29

**Authors:** Yingqi Nong, Qiyi Zhai, Wenjuan Liu, Jiahui Wei, Zhaoyi Wang, Xiaoyin Lv, Zitao Li, Xiqian Zhang, Fenghua Liu

**Affiliations:** ^1^ Department of Reproductive Health and Infertility Guangdong Women and Children Hospital Guangzhou Guangdong China; ^2^ Department of Traumatic Orthopedics, ZhuJiang Hospital Southern Medical University Guangzhou Guangdong China; ^3^ Graduate School of Guangzhou Medical University Guangzhou Guangdong China

**Keywords:** aquaporin 3, IGF2BP1, METTL14, PI3K/AKT, trophoblast migration and invasion

## Abstract

Reduced trophoblast migration and invasion contribute to unexplained recurrent spontaneous abortion (URSA). Aquaporin 3 (AQP3) plays a crucial role in facilitating trophoblast migration and invasion during early pregnancy through fetal‐maternal crosstalk. This study aimed to comprehensively investigate the mechanism involving AQP3 and its modulatory effects on human extravillous trophoblast (HTR‐8/SVneo cells) migration and invasion. AQP3 and IGF2BP1 expression was analysed using immunohistochemistry and quantitative real‐time polymerase chain reaction. The AQP3‐associated molecular mechanisms were explored using western blot, meRIP, RNA stability assays and RNA‐protein pull‐down experiments. Furthermore, the role of IGF2BP1 in HTR‐8/SVneo cells was assessed using transwell assays. AQP3 and IGF2BP1 expression was lower in the chorionic villi samples of the URSA group than in those of the control group. AQP3 was involved in regulating the activation of the PI3K/AKT signalling pathway. Additionally, METTL14 interacted with AQP3 mRNA, thereby influencing its stability. Furthermore, AQP3 mRNA bound to the IGF2BP1 protein, and IGF2BP1 knockdown resulted in reduced AQP3 mRNA stability and impaired trophoblast migration and invasion. METTL14 and IGF2BP1 stabilise AQP3 mRNA expression by mediating m6A, thereby facilitating HTR‐8/SVneo cell migration and invasion via the PI3K/AKT signalling pathway. Targeting AQP3 could potentially contribute to strategies aimed at mitigating URSA development.

## 1 Introduction

Recurrent spontaneous abortion (RSA), characterised by more than three consecutive spontaneous abortions before 28 weeks of pregnancy, poses a serious threat to women's health worldwide [1]. The incidence of RSA is approximately 2.5% in women of reproductive age [2]. Various factors, including cytogenetic abnormalities, anatomic irregularities, endocrine disorders, infections, autoimmunity, atypical blood clotting, sperm quality and environmental factors, may contribute to miscarriage. Moreover, approximately 50% of RSA cases are classified as unexplained recurrent spontaneous abortion (URSA), and the treatment for this condition remains controversial. Insufficient trophoblast cell migration and invasion are the primary causes of URSA [3, 4]. The invasive process is executed by extravillous trophoblast cells (EVTs), which differentiate from the cytotrophoblast cells of the blastocyst and subsequently undergo fusion to form syncytiotrophoblasts. However, the mechanisms regulating EVTs migration and invasion remain poorly understood.

Aquaporins (AQPs) are currently the only known proteins capable of mediating the transmembrane transport of water molecules [5]. We have previously demonstrated that AQP3 is highly expressed on the membrane of mouse blastocyst trophoblastic cells [6]. According to previous studies [7, 8], AQP3 is abundant in normal placentas and localised to the apical membrane of syncytiotrophoblast cells. Moreover, AQPs mediate the transmembrane transport of small molecules and promote cell migration and invasion [9, 10, 11, 12, 13]. Although our previous findings suggest that AQP3 promotes trophoblast cell migration and invasion possibly via the activation of the phosphoinositide 3‐kinase (PI3K)/AKT signalling pathway [14, 15], these results have not yet been validated.

In most eukaryotic mRNAs, N6‐methyladenosine (m6A) formation is the most prevalent and important post‐transcriptional epigenetic modification [16]. Currently, three classes of proteins involved in m6A methylation have been identified: methyltransferases, demethylases and methyl‐binding proteins, also known as ‘writer’, ‘eraser’ and ‘reader’ proteins, respectively [17]. Notably, a previous study has demonstrated that the m6A methylation level of global mRNA is significantly reduced in placental villus tissue from patients with RSA, and that m6A demethylase ALKBH5 can inhibit the invasion of trophoblast cells by regulating the stability of *CYR61* mRNA [18]. These results suggest the important role of m6A methylation in the migration and invasion of trophoblast cells at the maternal–fetal interface.

The presence of m6A methylation modification sites in AQP3 mRNA has been predicted using data from the SRAMP website. Bioinformatics analysis and literature review indicated that AQP3 mRNA is likely to be recognised by the m6A writer proteins methyltransferase‐like 13 (METTL3), METTL14 and Wilms' tumour 1‐associated protein (WTAP). Additionally, the insulin‐like growth factor 2 mRNA‐binding protein 1 (IGF2BP) family (including IGF2BP1–3), a newly identified group of m6A reader proteins, can enhance target mRNA stability and translation by recognising m6A modifications on target mRNAs [19, 20, 21]. We investigated the potential binding site between AQP3 mRNA and IGF2BP1 protein on the RM2Target website and suggested the presence of a binding interaction. Therefore, in the present study, we aimed to investigate whether AQP3 could bind to METTL3, METTL14, WTAP and IGF2BP1, and elucidate the nature of their interactions with each other.

## 2 Materials and Methods

### 2.1 Clinical Sample Collection

The chorionic villi samples (CVS) of 26 patients diagnosed with URSA were collected at 6–10 weeks of pregnancy, totalling 26 samples. The inclusion criteria were as follows: (1) age, 20–38 years old; (2) two or more consecutive spontaneous abortions before 28 weeks of gestation with the same spouse; (3) previous abortion history excluded embryo chromosome abnormality. The exclusion criteria were as follows: (1) either of the couples had chromosomal abnormalities or genetic diseases that could lead to abortion; (2) patients with uterine malformation and abnormal uterine cavity; (3) patients with endocrine disorders, such as thyroid disease, diabetes and polycystic ovary syndrome; (4) patients with severe cardiovascular disease, abnormal liver function, tumour, etc. that cannot tolerate pregnancy; (5) patients with abnormal immune function; (6) patients with severe infection during pregnancy; (7) prothrombotic state. For the control group, the CVS of 18 healthy women who underwent induced abortion at 6–10 weeks of pregnancy for personal reasons was used, yielding a total of 18 samples. The inclusion criteria were: (1) age, 20–38 years old; (2) patient induced abortion for personal reasons; (3) embryo development was normal before abortion. The exclusion criteria were the same as those of the experimental group. Both groups were excluded with chromosomal abnormalities.

### 2.2 Immunohistochemical (IHC) Analysis

IHC staining was performed using a DAB kit (Cat No. AR1027; Boster Biological Technology Co. Ltd., Wuhan, China) and a rabbit two‐step detection kit (Cat No. SV0002; Boster Biological Technology Co. Ltd.) according to manufacturer instructions to analyse the expression of AQP3 and IGF2BP1. For each IHC section, six fields were selected in the same standard, with an emphasis on including as many EVTs as possible, while minimising the presence of cytotrophoblast and syncytiotrophoblast cells. In the URSA cohort, 26 CVS samples were initially collected; however, EVTs were not detected in the tissue sections of 5 samples, resulting in the utilisation of only 21 URSA samples for subsequent integrated optical density (IOD) analyses. In contrast, EVTs were identified in the tissue sections of 18 control cases. IHC without the primary antibody was carried out as a negative control which was included in each run. Under light microscopy, the cells within the field of view exhibited brownish‐yellow particles, indicating positivity for AQP3 and IGF2BP1. Quantification of IHC staining was performed using Image‐Pro Plus v6.0 software following the Image User Guide, and then selecting Area of Interesting (AOI) and IOD to gain Mean of IOD (IOD/AOI, MI).

### 2.3 Cell Line, Plasmids and Transfection

The human trophoblast cell line HTR‐8/SVneo was purchased from the American Type Culture Collection (Manassas, VA, USA) and grown in Dulbecco's modified Eagle's medium (DMEM, Invitrogen, Waltham, MA, USA) containing 10% fetal bovine serum (FBS) and 1% penicillin–streptomycin. Cells were cultured at 37°C in a 95% air and 5% CO_2_ atmosphere. Plasmids containing vectors for overexpression (OE) or knockdown of AQP3 and IGF2BP1 were purchased from IGEbio (Guangzhou, China). Empty interfering (CON‐shRNA) and overexpressing (CON‐OE) vectors were used as negative controls. Plasmids were transfected into the cells using Lipofectamine 3000 (Invitrogen) according to the manufacturer's instructions.

For the gene knockdown experiments in HTR‐8/SVneo, three siRNA sequences targeting METTL3, METTL14 and WTAP were designed and synthesised. HTR8 cells were seeded at 5 × 10^4^ cells per well in 24‐well plates and transfected with siRNAs at a final concentration of 50 nM using Lipofectamine RNAiMAX. Following a 6‐h incubation period with the transfection complexes, the medium was replaced with a fresh complete growth medium. After 48 h, the knockdown efficiency was assessed by qPCR, selecting the most effective sequence for each gene.

The experiments were performed in triplicate to ensure reproducibility.

### 2.4 Western Blot

The expression levels of the proteins PI3K, phosphorylated PI3K (P‐PI3K), AKT and phosphorylated AKT (P‐AKT) were assessed in the AQP3‐overexpression (AQP3‐OE), control overexpression (CON‐OE), AQP3‐shRNA, control shRNA (CON‐shRNA) and AQP3‐OE combined with LY294002 treatment groups. LY294002 is a specific inhibitor of PI3K. HTR8 cells with stable overexpression of AQP3 were treated with LY294002 (Cell Signalling Technology, #9901) by exposure to 10 μM of the inhibitor for 1 h prior to cell harvesting.

Cells were harvested at the indicated times and rinsed twice with ice‐cold phosphate‐buffered saline. Cell extracts were prepared using lysis buffer and centrifuged at 13,000 × *g* for 10 min at 4°C. Protein samples were electrophoresed in 12% polyacrylamide gels and transferred to polyvinylidene fluoride membranes. The samples were blocked using 5% skimmed milk powder for 1 h at room temperature (20°C–25°C) and then incubated with primary antibodies, namely PI3K (Abcam, #ab180967, 1:1000), p‐PI3K (Bioworld, #BS4605, 1:1000), AKT (Affinity, #AF0836, 1:1000), p‐AKT (CST, #4060S, 1:1000) and GAPDH (Aksomics, #KC‐5G4, 1:5000), in Tris‐buffered saline with Tween‐20 overnight at 4°C. Horseradish peroxidase‐conjugated secondary antibodies (Boster, BA1050, 1:10,000) were used to label the primary antibodies. After the horseradish peroxidase substrate was added to the membranes, the signals were examined using autoradiography films. All Western blot analyses were repeated three times.

### 2.5 Transwell Migration and Invasion Assays

Cell migration and invasion were assessed using a transwell assay. For migration assays, Matrigel (1:8) (BD Biosciences, Bedford, MA, USA) was diluted with serum‐free DMEM, and the basement membrane of the upper chamber of the transwell was coated. The solution was kept at 37°C for 1–4 h to transform the Matrigel aggregate into the gel. Treated cells were harvested and dilution with serum‐free DMEM (5 × 10^5^ cells/mL) 200 μL was added to a transwell insert (pore size, 8 μm; BD Biosciences, San Jose, CA, USA), and 600 μL containing 20% FBS was added to the lower chamber. Cells at each concentration were cultured in a 24‐well plate in a 5% CO2 incubator at 37°C for 24 h. The culture medium in each well was then discarded, and the chamber was washed twice with PBS. After gently removing the cells in the upper chamber with a cotton swab, the cells on the underside of the membrane were fixed with 4% paraformaldehyde for 15 min, stained with 0.1% cresyl violet, washed three times with PBS, and air‐dried. Five fields (200 × magnification) were randomly selected to count the number of migrated cells, and images were taken by using phase contrast microscopy. Transwell invasion assays were performed as previously described [15]. Transwell migration and invasion assays were performed in triplicate.

### 2.6 RNA Stability Assays

IGF2BP1‐knockdown, IGF2BP2‐OE and corresponding control cell lines were cultured in six‐well plates. Actinomycin D (5 mg/mL) was then added at 0, 2, 4, 6 or 8 h before scraping and collecting the cells. Total RNA was isolated using TRIzol reagent, and qRT‐PCR was performed to quantify the relative levels of AQP3 mRNA. HTR‐8/SVneo cells were treated with 100 μg/mL cycloheximide (CHX #HY‐12320; MedChemExpress, Monmouth Junction, NJ, USA) for 0, 4 or 8 h. The lysates were analysed using western blotting at different time points. All assays were repeated three times.

### 2.7 RNA Extraction and Quantitative Real‐Time Polymerase Chain Reaction (qRT‐PCR)

Total RNA was extracted from the cells using a TRIzol kit (Takara Bio, Kusatsu, Japan). First‐strand complementary DNA was generated from total RNA using an oligo‐dT/random primer mix and reverse transcriptase (Takara Bio). qRT‐PCR was conducted using the QuantStudio 5 Real‐Time PCR system (Thermo Fisher Scientific, Waltham, MA, USA), and the primers were designed using Primer‐BLAST (NCBI, Bethesda, MD, USA) (Table 1). The relative gene expression was normalised to endogenous GAPDH mRNA expression detected in each experimental sample. The fold changes were calculated using the 2^−ΔΔC^_T_ method according to the manufacturer's instructions. All reactions were run in triplicate.

**TABLE 1:** Primer sequences and the product size of target and control genes.

Primer	Primer sequence (5′–3′)	Accession number	Size (bp)
GAPDH	Forward primer: ACTCCTCCACCTTTGACGCT	NM_002046.6	187
	Reverse primer: GGTCTCTCTCTTCCTCTTGTGC		
AQP3	Forward primer: ACCATCAACCTGGCCTTTGG	NM_004925.5	390
	Reverse primer: GGGGACGGGGTTGTTGTAG		
IGF2BP1	Forward primer: AAGGGGGCCATCGAGAATTG	NM_006546.4	222
	Reverse primer: GGGAGCCTGCATAAAGGAGC		
METTL3	Forward primer: CAGGGCTGGGAGACTAGGAT	NM_019852.5	172
	Reverse primer: CTGGGCTGTCACTACGGAAG		
METTL14	Forward primer: GGTTGGACCTTGGAAGAGTGT	NM_020961.4	249
	Reverse primer: TGCCAATTTCAGGTTCTTCTGTG		
WTAP	Forward primer: ATGGCGAAGTGTCGAATGCT	NM_004906.5	77
	Reverse primer: AGTTGTGCAATACGTCCCTGG		
B‐Actin	Forward primer: AGCATCCCCCAAAGTTCACAAT	NM_001101.5	127
	Reverse primer: AGTGGGGTGGCTTTTAGGATG		

### 2.8 Cell Climbing–Fluorescence Probe‐FISH and Immunofluorescence Protocol

The RNA fluorescence in situ hybridisation was performed using a probe against AQP3 RNA (BersinBio, #QD792) in accordance with the protocol of the manufacturer for the simultaneous FISH and immunofluorescence. Briefly, cells were fixed in 4% paraformaldehyde for 20 min, washed thrice with PBS and delineated using a liquid blocker pen before Proteinase K digestion. Hybridisation involved prehybridisation at 37°C for 1 h, followed by overnight incubation with a AQP3‐CY3 probe (BersinBio, #QD792, 3:100). Washing was conducted using 2 × SSC, 1 × SSC, and 0.5 × SSC steps. Blocking utilised blocking serum, and METLL14 antibody (Proteintech, #26158‐1‐AP) incubations were performed sequentially with primary and secondary antibodies (Servicebio, #GB25303), followed by DAPI staining. Fluorescence microscopy was employed for visualisation with specific wavelength settings for DAPI, FAM(488) and CY3. DAPI appears blue, FAM appears green, CY3 appears red. Analysis confirmed cytoplasmic and nuclear localisation of signals, indicating miRNA and lncRNA variability. All procedures ensured an RNase‐free environment to maintain RNA integrity, aligning with protocols suitable for publication in the Journal of Cellular and Molecular Medicine. The experiment was repeated three times.

### 2.9 RNA‐Protein Pull‐Down Analyses

Transfected and induced cells were harvested, and cell lysates were prepared using RNA immunoprecipitation (RIP) lysis buffer (Huijun Biotechnology, Guangzhou, China). AQP3 and its mutant versions were transcribed using a T7 in vitro transcription kit (Invitrogen) and labelled in vitro using an RNA 3′‐end desthiobiotinylation kit (QIAGEN, Hilden, Germany). Beads (50 μL) and labelled RNA (50 pmol) were added to the RNA capture buffer and incubated for 30 min at room temperature (20°C–25°C) with agitation to bind labelled AQP3 mRNA to the streptavidin magnetic beads (Thermo Fisher Scientific). After washing the beads with an equal volume of 20 mM Tris (pH 7.5), 100 μL of the protein‐RNA‐binding buffer was added and thoroughly mixed with the beads. The master mix of the RNA‐protein binding reaction (100 μL) was added to the RNA‐bound beads, mixed by pipetting and then incubated for 60 min at 4°C with rotation to allow the binding of RNA‐binding proteins to RNA. After washing the beads twice with 100 μL of wash buffer, 50 μL of elution buffer was added and incubated for 30 min at 37°C with agitation. The samples were then run on a gel and analysed. Each pulldown was repeated in triplicates.

### 2.10 RIP Assay

Cells were harvested, washed and incubated with ice‐cold RIP lysis buffer (Huijun Biotechnology) containing 0.5% ribonuclease inhibitor (QIAGEN) on ice for 5 min with periodic mixing. Subsequently, the lysates were transferred into a microcentrifuge tube and centrifuged at 13,000 × *g* for 10 min at 4°C to pellet the cell debris. The supernatants were transferred into a fresh tube, and protein G agarose was added and incubated for 1 h at 4°C with rotation for pre‐clearing. The immunoprecipitating antibody was added and incubated overnight at 4°C with rotation. Protein G agarose was pelleted using brief centrifugation (3000 × *g* for 1 min) and then washed sequentially with the immunoprecipitation (IP) lysis buffer (containing 0.5% ribonuclease inhibitor). Finally, the RNA was extracted from protein/RNA complexes on the beads using TRIzol reagent, dissolved in diethyl pyrocarbonate‐treated water, and quantified using quantitative PCR (qPCR). Experiments were repeated three times.

### 2.11 Methylated RNA Immunoprecipitation (MeRIP) Assay

The MeRIP assay was performed according to the MeRIP N6‐methyladenosine (m6A) kit (BersinBio, Bes5203‐2) procedure. The magnetic beads (A/G blend; 25 μL) were washed and incubated with an anti‐m6A antibody (5 μL) at room temperature (20°C–25°C) for 30 min and washed with PBS three times to remove unbound antibody molecules. Total RNA (15 μg) was extracted from the HTR‐8/SVneo cells. The RNA samples were treated at 85°C for 5 min, immediately chilled on ice and then incubated with m6A antibody‐associated beads for 2 h at 4°C with rotation. The RNA‐associated magnetic beads were then washed three times. Total RNA was extracted from the beads using TRIzol reagent and quantified using qRT‐PCR. The assay was repeated three times.

### 2.12 Statistical Analysis

Two‐sided Student's *t*‐test, one‐way analysis of variance (ANOVA) and Bonferroni's multiple comparison test were used to determine the statistical significance of group effects. Statistical analyses were performed using GraphPad Prism 8.0 software (GraphPad, San Diego, CA, USA). A 95% confidence interval was considered significant and defined as *p* < 0.05.

## 3 Results

### 3.1 Expression of AQP3 and IGF2BP1 in the CVS of Patients With URSA and Control Groups

**FIGURE 1:**
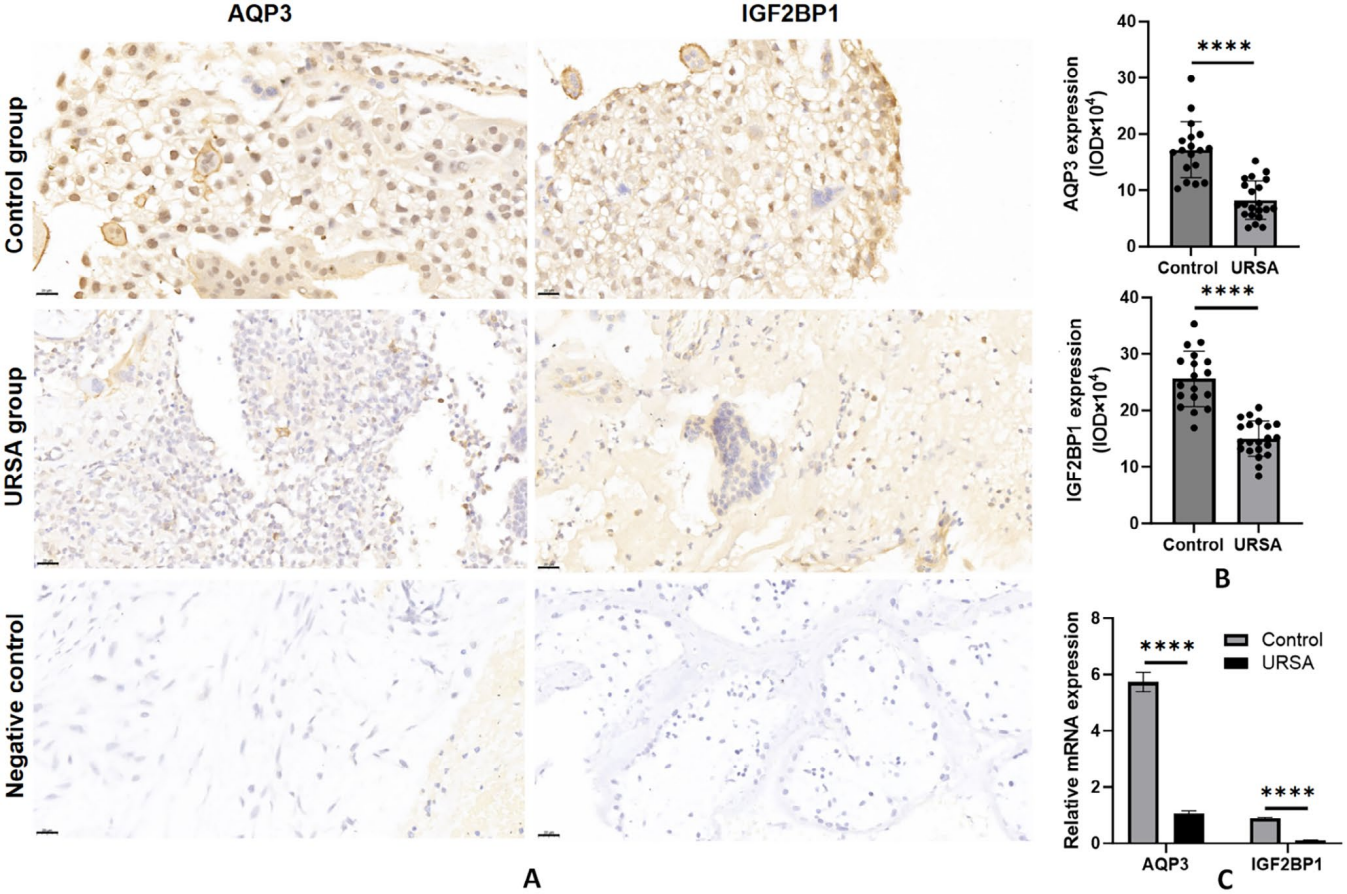
The expression of AQP3 and IGF2BP1 in the CVS obtained from patients with URSA compared to a control group. (A) IHC staining shows AQP3 and IGF2BP1 localisation in EVTs of URSA and control groups at ×400 magnification. Scale bar = 20 μm. (B) Comparison of MI of AQP3 and ICF2BP1 in EVTs between URSA (*n* = 21) and control (*n* = 18) groups. (C) Polymerase chain reaction analysis of the mRNA expression level of AQP3 and IGF2BP1 in the CVS of URSA (*n* = 26) and control (*n* = 18) groups. Each plotted value corresponds to the mean ± SEM. ****p < 0.0001. AQP3, aquaporin 3; CVS, chorionic villi samples; EVTs, extravillous trophoblast; IGF2BP, insulin‐like growth factor 2 mRNA‐binding protein 1; IHC, immunohistochemical; MI, mean of integrated optical density; URSA, unexplained recurrent spontaneous abortion.

IHC staining was conducted on the CVS of the URSA and control groups to analyse the expression of AQP3 and IGF2BP1. AQP3 and IGF2BP1 were detected in EVTs of both URSA and control groups (Figure 1A). The results of IHC semi‐quantitative analysis of IOD for AQP3 and IGF2BP1 indicated a significant decrease in their expression levels in the EVTs of URSA compared to those in the control group (*p* < 0.0001). In the URSA (*n* = 21) and control (*n* = 18) groups, the MI for AQP3 in EVTs cells were observed to be 8.24 ± 3.43 and 17.21 ± 4.99, respectively, while the MI for IGF2BP1 were 14.98 ± 3.13 and 25.59 ± 4.93, respectively (Figure 1B).

Additionally, compared to the control group, the mRNA expression levels of AQP3 in the URSA group of the CV and decidua tissue decreased by 81.53%, while the mRNA expression levels of IGF2BP1 decreased by 86.68% (*p* < 0.0001, Figure 1C).

### 3.2 AQP3 May Enhance Trophoblast Migration and Invasion Through Activating the PI3K/AKT Pathway

**FIGURE 2:**
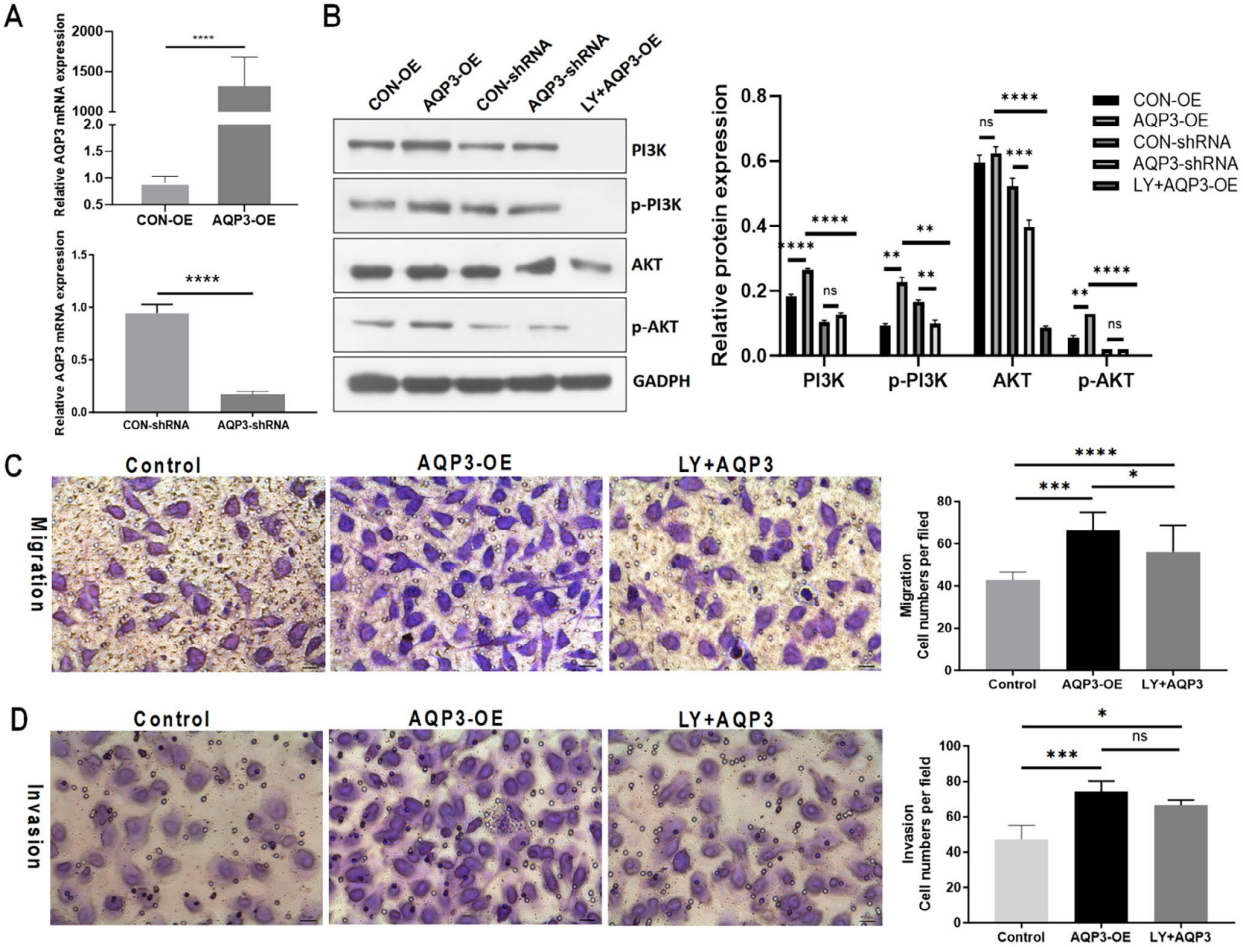
AQP3 may enhance trophoblast migration and invasion by activating the PI3K/AKT pathway. (A)Relative expression levels of AQP3 mRNA in AQP3‐OE HTR8/SVneo and AQP3‐shRNA HTR8/SVneo cells. (B) Western blot analysis of the PI3K/AKT signalling pathway in the CON‐OE, AQP3‐OE, CON‐shRNA, AQP3‐shRNA and LY294002 + AQP3 groups. Cell migration (C) and invasion (D) capacities in the AQP3‐OE, AQP3‐OE + LY294002, and Control groups. Scale bar = 25 μm. Representative photographs of migratory or invasive cells (magnification, ×200) are shown. Each plotted value corresponds to the mean ± SEM. LY:LY294002. *p < 0.05, ***p < 0.001. Bars represent mean values and error bars represent SD. AKT, protein kinase B; AQP3, Aquaporin 3; CON, control; OE, overexpression; PI3K, phosphoinositide 3‐kinase; shRNA, short hairpin RNA.

Following the construction of stable cell lines with AQP3 OE or AQP3 knockdown, the knockdown and OE efficiency were verified using qPCR. The results indicated that compared to the CON‐OE group, AQP3 OE resulted in the upregulation of AQP3 mRNA levels by 1441.31‐fold (*p* < 0.0001; Figure 2A).

Western blotting was used to compare PI3K, p‐PI3K, AKT and p‐AKT expression levels among the CON‐OE, AQP3‐OE, CON‐shRNA, AQP3‐shRNA and LY294002 + AQP3 groups. The expression levels of PI3K, p‐PI3K and p‐AKT were significantly upregulated in the AQP3‐OE group compared to those in the CON‐OE group (*p* < 0.0001, *p* = 0.0082 and *p* = 0.0068, respectively). Conversely, AQP3 downregulation resulted in a significant decrease in the expression levels of p‐PI3K and AKT (*p* = 0.0082, *p* = 0.0006, respectively). Furthermore, the addition of the PI3K inhibitor LY294002 to AQP3‐OE resulted in significantly lower expression levels of PI3K, p‐PI3K, AKT and p‐AKT compared to those in the AQP3‐OE group (*p* < 0.0001, *p* = 0.0045, *p* < 0.0001 and *p* < 0.0001, respectively; Figure 2B).

Transwell invasion assay results indicated that the migration and invasion abilities of the cells in the AQP3‐OE group were upregulated by 90.21% and 56.96%, respectively, compared to those in the CON‐OE group (*p* < 0.001). After the addition of the PI3K/AKT signalling pathway inhibitor LY294002 to AQP3‐OE cells, these abilities increased to a lesser extent, by 61.47% (*p* < 0.05) and 40.93% (*p* < 0.001), respectively, compared to their corresponding levels in the control group (Figure 2C,D).

### 3.3 METTL14 m6A Methyltransferase Seems to Affect AQP3 mRNA Stability

**FIGURE 3:**
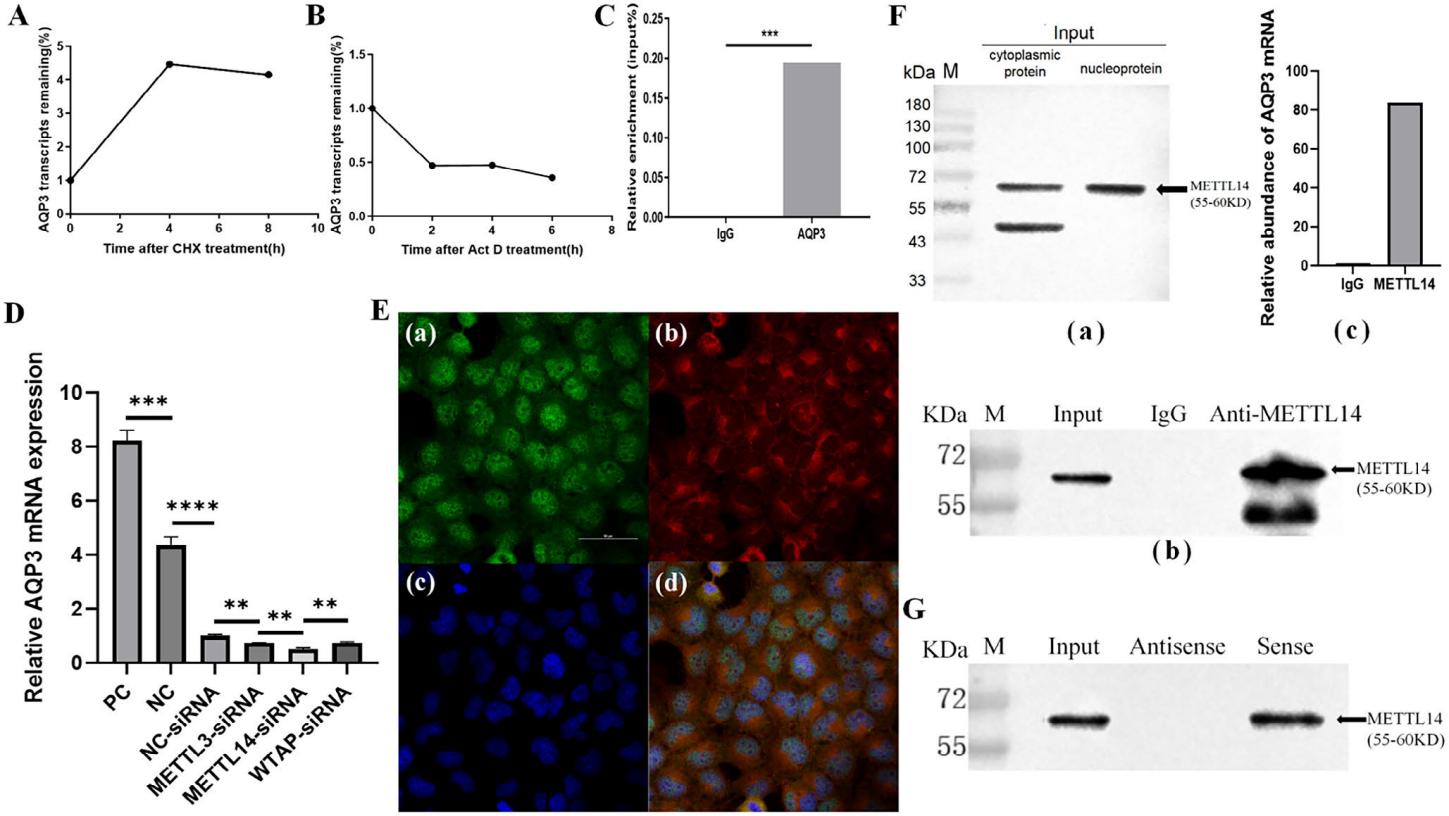
The stability of AQP3 mRNA is regulated by the m6A methyltransferase METTL14. (A) Actinomycin D assay to assess the stability of AQP3 mRNA. (B) Cycloheximide assay to assess the protein stability of AQP3. (C) meRIP assay analysed m6A‐modified AQP3 enrichment in HTR‐8/SVneo cells. The enrichment of RIP was normalised by input RNA via qRT‐PCR. IgG group, Negative control; IP group, Untreated HTR‐8/SVneo cells were utilised in immunoprecipitation assays. (D) AQP3 mRNA expression levels following the knockdown of METTL14, METTL3 and WTAP. PC, The positive control group; NC, The negative control; NC‐siRNA, The negative control group transfected with siRNA. Each plotted value corresponds to the mean ± SEM. (E) FISH/IF analysis of the colocalisation of METTL14 and *AQP*3 mRNA in the nuclear membranes: (a) METTL14 (green fluorescence); (b) AQP3 (red fluorescence); (c) Nucleus (blue fluorescence); (d) Composite image. Scale bar = 50 μm. (F) RIP‐qPCR detection of METTL14 and AQP3 mRNA binding to each other: (a) The RNA immunoprecipitation assay identified METTL14 protein in the cytoplasm and nucleus. (b) RIP/WB detection of METTL14‐RNA complex in nucleus Input. (c) PCR analysis of AQP3 mRNA expression levels in the two groups of complexes. (G) RNA‐pull‐down assay showing an interaction between METTL14 and AQP3 mRNA. **p < 0.01, ***p < 0.001, ****p < 0.0001. AQP3, aquaporin 3; FISH‐IP, fluorescence in situ hybridizationhybridisation‐immunoprecipitation; HTR‐8/SVneo, human extravillous trophoblast cell line; IgG, immunoglobulin Gl; IP, immunoprecipitation; m6A, N6‐methyladenosine; METTL14, methyltransferase‐like 14; METTL3, methyltransferase‐like 3; PCR, polymerase chain reaction; RIP, RNA immunoprecipitation; WB, western blot; WTAP, Wilms' tumourtumor 1‐associated protein.

Actinomycin D and CHX assays were performed to determine the stability of AQP3 mRNA and protein, respectively. The stability of AQP3 mRNA diminished as the incubation duration of HTR‐8/SVneo cells with Actinomycin D was extended (Figure 3A). In contrast, AQP3 protein synthesis in HTR‐8/SVneo cells remained constant irrespective of the duration of incubation with CHX (Figure 3B). Moreover, qPCR was utilised to assess the expression levels of AQP3 mRNA in the MeRIP assay products, revealing enrichment of AQP3 mRNA in the IP group (0.1944) compared to the total protein samples (*p* < 0.001; Figure 3C).

Following the knockdown of METTL14, METTL3 and WTAP in HTR8 cells, the AQP3 mRNA levels decreased. Notably, the reduction in AQP3 mRNA levels was the most pronounced in the METTL14 knockdown group, with significant differences observed compared to the other experimental groups (Figure 3D).

FISH‐IP assay indicated that METTL14 and AQP3 mRNA were co‐localised in the cytoplasm and nuclear membranes (Figure 3E). For the RIP experiment, we analysed both the cytoplasmic and the nuclear proteins, discovering that the binding sites of the two molecules within both cytoplasmic and nuclear compartments (Figure 3Fa), The analysis of the cytoplasmic protein revealed two distinct bands: one corresponding to METTL14 and the other representing an unidentified protein, likely an alternative state of METTL14. In contrast, the nuclear protein exhibited a single band, corresponding to METTL14. For further analysis, nuclear proteins were chosen for subsequent q‐PCR assays and RNA pull‐down experiments. The bait protein METTL14 was utilised to isolate the METTL14‐RNA complex (Figure 3Fb). AQP3 exhibited enrichment by approximately 83.6‐fold compared to IgG in the METTL14 group (Figure 3Fc). Moreover, to identify proteins capable of binding to AQP3 mRNA, RNA‐pull‐down with protein spectrum analysis was used. The control sample utilised the AQP3 RNA antisense strand. METTL14 protein bands were detected in both the input and experimental groups, whereas no METTL14 protein band was observed in the control group, indicating an interaction between *AQP3* RNA and METTL14 protein (Figure 3G).

### 3.4 AQP3 mRNA Binding to IGF2BP1

**FIGURE 4:**
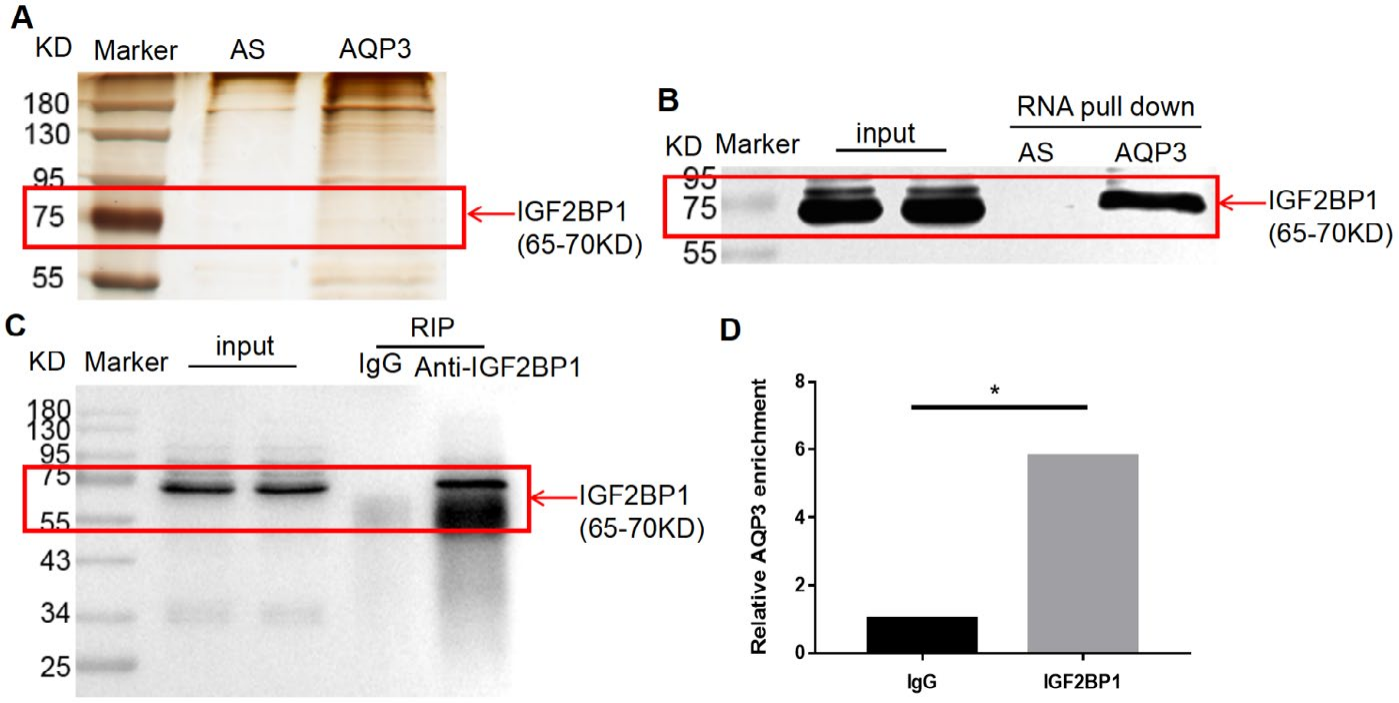
Binding of AQP3 mRNA to IGF2BP1. (A, B) RNA pull‐down and western blot assays were used to identify the binding between the IGF2BP1 protein and AQP3 mRNA in HTR‐8/SVneo cells. AS: Samples obtained from the antisense RNA strand of AQP3 (control group). AQP3: Complexes obtained from the sense RNA strand of AQP3 (experimental group). (C, D) An RNA immunoprecipitation assay was performed using an anti‐IGF2BP1 or control IgG antibody followed by PCR analysis of AQP3 mRNA enrichment. IgG group: Negative control. *p < 0.05. AQP3, aquaporin 3; IGF2BP1, insulin‐like growth factor 2 mRNA‐binding protein 1; IgG, immunoglobulin G.

The RNA pull‐down assay was used to identify the proteins that bind to AQP3 mRNA. The complexes obtained from the AQP3 sense RNA strand served as samples for the experimental (AQP3) group, whereas samples obtained from the AQP3 antisense RNA strand served as controls (AS group; Figure 4A). The IGF2BP1 protein bands were detected in the input and experimental groups but not in the control group (Figure 4B), indicating interactions between AQP3 mRNA and IGF2BP1 protein.

In the RIP assay, the IGF2BP1 and IgG groups served as the experimental and RIP‐negative control groups, respectively. The input represented the total cellular protein, with an anti‐IGF2BP1 antibody used to detect the products of the RIP assay. When the IGF2BP1 protein was used as bait, the western blot results were positive, indicating that IGF2BP1‐RNA complexes were successfully obtained in the assay (Figure 4C). After normalising the data from the input group, AQP3 mRNA in the IGF2BP1 group was enriched by 0.28% compared to that in the input group. Normalisation of the IgG group data revealed that AQP3 mRNA in the IGF2BP1 group was enriched by approximately 5.8‐fold compared to that in the IgG group (Figure 4D). These results indicate interactions between the IGF2BP1 protein and AQP3 mRNA.

### 3.5 IGF2BP1 Knockdown Inhibits, Whereas IGF2BP1 OE Promotes Cell Migration and Invasion

**FIGURE 5:**
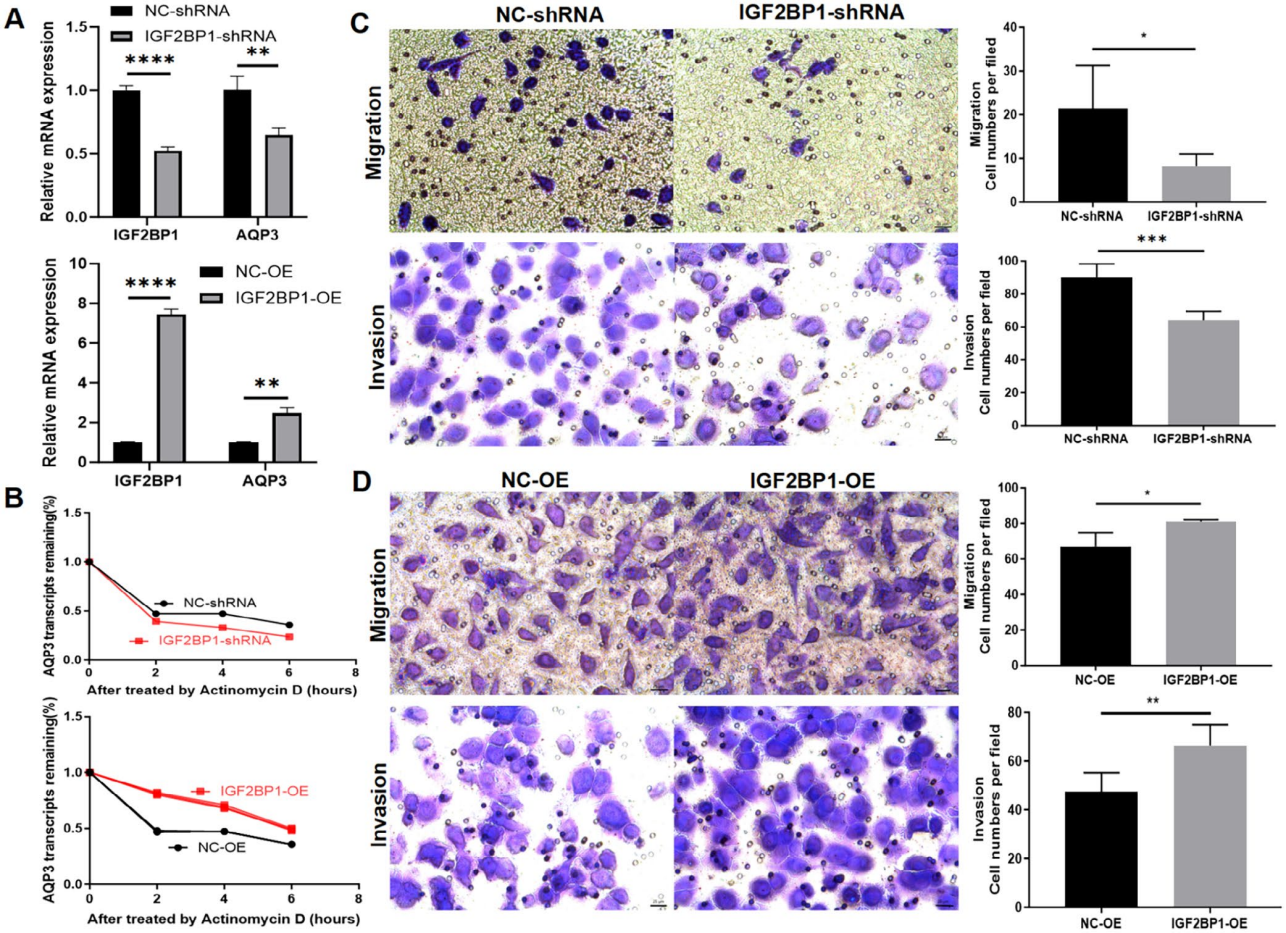
The impact of modulating IGF2BP1 expression on the migratory and invasive properties of HTR‐8/SVneo cells. (A) qPCR analysis of *IGF2BP1* knockdown and OE efficiencies at the IGF2BP1 *and* AQP3 mRNA level in HTR8/Svneo cells. (B) Actinomycin D assay was conducted following IGF2BP1 knockdown or overexpression. (C) IGF2BP1 knockdown led to a decrease in cell migration and invasion ability. (D) IGF2BP1 overexpression enhanced cell migration and invasion ability. Representative photographs of migratory or invasive cells (magnification, ×200) are shown. Scale bar = 25 μm. Each plotted value corresponds to the mean ± SEM. *p < 0.05, **p < 0.01, ***p < 0.001, ****p < 0.0001. AQP3, aquaporin 3; IGF2BP1, insulin‐like growth factor 2 mRNA‐binding protein 1; NC, negative control group; OE, overexpression.

RT‐PCR results showed that IGF2BP1 knockdown reduced IGF2BP1 mRNA levels by 47.51% and AQP3 mRNA levels by 56.06%, compared to the CON‐shRNA group. Conversely, IGF2BP1 overexpression increased IGF2BP1 mRNA levels by 7.45‐fold and AQP3 mRNA levels by 2.496‐fold (Figure 5A).

The actinomycin D assay indicated that IGF2BP1 downregulation in HTR‐8/SVneo cells resulted in a decrease in the stability of AQP3 mRNA, whereas its overexpression resulted in an increase in the stability of AQP3 mRNA (Figure 5B).

Transwell assay results revealed the migration and invasion abilities of cells in the IGF2BP‐ knockdown group were reduced by 61.68% (*p* < 0.001) and 29.05% (*p* < 0.05), respectively, compared to those in the negative control group (Figure 5C). In contrast, the migration and invasion rates were higher in the IGF2BP1‐OE cells than those in the CON‐OE groups by 20.70% (*p* < 0.05) and 39.66% (*p* < 0.01), respectively (Figure 5D).

## 4 Discussion

A previous study [22] identified AQP3 as the most abundant AQP expressed in chorionic villi samples from the first trimester, and downregulation of AQP3 expression significantly attenuates trophoblast cell migration [10]. In our study, AQP3 was observed in EVTs of both the URSA and control groups. Moreover, our IHC semi‐quantitative IOD analysis of AQP3 and qPCR revealed significantly lower protein and mRNA expression levels of AQP3 in the URSA of the CV and decidua tissue than those in healthy pregnant women. Therefore, we further analysed the molecular mechanisms acting upstream and downstream of AQP3 to regulate the migration and invasion of human extravillous trophoblast cells.

The PI3K/AKT signalling pathway is one of the major signalling pathways that regulate trophoblast function at the maternal–fetal interface. It plays a positive regulatory role in trophoblast cell invasion, migration, proliferation and differentiation during early pregnancy [23, 24, 25]. In the present study, we observed that the overexpression of AQP3 led to an increase in p‐PI3K and p‐AKT levels, whereas its knockdown resulted in a decrease in p‐PI3K and p‐AKT levels. Moreover, treatment with LY294002, an inhibitor of the PI3K/AKT signalling pathway, partially reversed the enhanced migratory and invasive properties induced by AQP3 overexpression. The data presented herein suggest that the PI3K/AKT signalling pathway may exert regulatory effects on trophoblast cell migration and invasion via modulation of AQP3 expression. It is imperative to acknowledge, however, that the PI3K/AKT pathway is also implicated in the regulation of cellular proliferation. This overlap in functions may account for some of the phenotypic outcomes observed in the current study. Consequently, while our findings lend support to the hypothesis that AQP3 plays a role in the dynamics of trophoblast cells mediated by PI3K/AKT signalling, additional investigations are warranted in the future to delineate the specific contributions of this pathway to cell migration and invasion distinct from its role in proliferation.

The m6A modification influences mRNA fate and plays a crucial role in many major biological processes [26]. In the present study, we observed a significant enrichment of m6A‐modified AQP3 mRNA in HTR‐8/SVneo cells. The results of the CHD and CHX assays suggested that the stability of AQP3 mRNA expression is greater than that of AQP3 protein expression. These observed alterations in mRNA expression levels are likely attributed to enhanced mRNA stability rather than increased mRNA expression. Moreover, the knockdown of METTL14, an m6A writer, exerted the most significant impact on reducing AQP3 levels in HTR8 cells. We further validated the interaction between METTL14 and AQP3 mRNA through FISH‐IP, RIP and RNA‐pull‐down experiments, confirming that METTL14 plays a role in regulating the stability of AQP3 mRNA in trophoblast cells.

IGF2BP1, as a m6A reader protein, likely binds to AQP3 mRNA via its m6A methylated sites, thereby promoting AQP3 mRNA stability. In this study, we observed IGF2BP1 expression in both EVTs within the CVS of both the URSA and control groups. Moreover, IHC semi‐quantitative IOD analysis of IGF2BP1 and qPCR revealed significantly lower protein and mRNA expression levels of IGF2BP1 in the URSA of the CVS than those in healthy pregnant women. We performed an RNA pull‐down assay to identify proteins that bind to AQP3 mRNA and a subsequent western blot analysis, confirming that AQP3 mRNA binds to IGF2BP1. RIP and PCR assays demonstrated an interaction between IGF2BP1 protein and AQP3 mRNA. Notably, IGF2BP1 knockdown was accompanied by lower AQP3 mRNA stability, which inhibited cell migration and invasion capabilities. In contrast, the opposite effects were noted after IGF2BP1 OE. The MeRIP and qPCR assays confirmed that AQP3 mRNA with m6A methylation sites was significantly enriched in HTR‐8/SVneo cells. The K homology domains of IGF2BP1 are responsible for recognising and binding to RNA sequences with m6A methylation marks, suggesting that the m6A reader function of IGF2BP1 promotes AQP3 mRNA stability. This may serve as a critical upstream mechanism underlying the effects of AQP3 on trophoblast migration and invasion.

In summary, our results demonstrated that METTL14 writes m6A modifications on AQP3 mRNA, with IGF2BP1 acting as a reader that binds to m6A modification on AQP3, thereby stabilising AQP3 mRNA to promote trophoblast cell migration and invasion by activating the PI3K/AKT signalling pathway. Thus, targeting AQP3 could potentially contribute to strategies aimed at mitigating URSA development, although it should be noted that the etiopathogenesis of URSA is multifactorial and complex. Further research is needed to fully understand the role of AQP3 within this broader context. However, the main limitation of this study is that the m6A methylation sites of AQP3 mRNA recognised by IGF2BP1 have not yet been identified. In the future, we intend to identify the m6A methylation sites of AQP3 mRNA recognised by IGF2BP1 at the molecular level, detect the key molecules involved in the expression of the components of the METTL14/IGF2BP1/AQP3/PI3K/AKT signalling axis in patients with URSA, ascertain the status of m6A methylation, analyse clinical correlations and assess the clinical transformation significance of the new molecular targets for URSA.

## Author Contributions

**Yingqi Nong:** project administration (equal), writing – original draft (lead). **Qiyi Zhai:** formal analysis (equal), methodology (equal), project administration (equal). **Wenjuan Liu:** data curation (equal), formal analysis (lead), methodology (equal). **Jiahui Wei:** data curation (equal), investigation (equal). **Zhaoyi Wang:** data curation (equal), investigation (equal), methodology (equal). **Xiaoyin Lv:** data curation (equal), methodology (equal). **Zitao Li:** investigation (equal), methodology (equal). **Xiqian Zhang:** supervision (lead), writing – review and editing (lead). **Fenghua Liu:** funding acquisition (lead), resources (lead), writing – original draft (supporting).

## Ethics Statement

This study was approved by the Ethics Committee of Guangdong Women and Children Hospital and has been performed in accordance with the principles of the Declaration of Helsinki.

## Consent

The patients/participants provided their written informed consent to participate in this study.

## Conflicts of Interest

The authors declare no conflicts of interests.

## Data Availability

The data that support the findings of this study are available on request from the corresponding author. The data are not publicly available due to privacy or ethical restrictions.
